# Rapid PCR Detection of *Mycoplasma hominis*, *Ureaplasma urealyticum*, and *Ureaplasma parvum*

**DOI:** 10.1155/2013/168742

**Published:** 2013-03-11

**Authors:** Scott A. Cunningham, Jayawant N. Mandrekar, Jon E. Rosenblatt, Robin Patel

**Affiliations:** ^1^Division of Clinical Microbiology, Department of Laboratory Medicine and Pathology, Mayo Clinic, Rochester, MN 55905, USA; ^2^Division of Biomedical Statistics and Informatics, Department of Health Science Research, Mayo Clinic, Rochester, MN 55905, USA; ^3^Division of Infectious Diseases, Department of Medicine, Mayo Clinic, Rochester, MN 55905, USA

## Abstract

*Objective.* We compared laboratory developed real-time PCR assays for detection of *Mycoplasma hominis* and for detection and differentiation of *Ureaplasma urealyticum* and *parvum* to culture using genitourinary specimens submitted for *M. hominis* and *Ureaplasma* culture. *Methods.* 283 genitourinary specimens received in the clinical bacteriology laboratory for *M. hominis* and *Ureaplasma* species culture were evaluated. Nucleic acids were extracted using the Total Nucleic Acid Kit on the MagNA Pure 2.0. 5 *μ*L of the extracts were combined with 15 *μ*L of each of the two master mixes. Assays were performed on the LightCycler 480 II system. Culture was performed using routine methods. *Results.* *M. hominis* PCR detected 38/42 *M. hominis* culture-positive specimens, as well as 2 that were culture negative (sensitivity, 90.5%; specificity, 99.2%). *Ureaplasma* PCR detected 139/144 *Ureaplasma* culture-positive specimens, as well as 9 that were culture negative (sensitivity, 96.5%; specificity, 93.6%). Of the specimens that tested positive for *Ureaplasma* species, *U. urealyticum* alone was detected in 33, *U. parvum* alone in 109, and both in 6. *Conclusion.* The described PCR assays are rapid alternatives to culture for detection of *M. hominis* and *Ureaplasma* species, and, unlike culture, the *Ureaplasma* assay easily distinguishes *U. urealyticum* from *parvum.*

## 1. Introduction

*Mycoplasma hominis*, *Ureaplasma urealyticum*, and *Ureaplasma parvum *are small, fastidious bacteria belonging to the Mollicutes class. They lack a cell wall (preventing staining with Gram stain) and are not sensitively detected on routine bacterial cultures. Optimal recovery requires specialized media and growth conditions. There are several human pathogens in the genera *Mycoplasma *and *Ureaplasma* which are responsible for a variety of clinical manifestations involving multiple body systems [1]. *M. hominis* causes septic arthritis and postpartum fever and has been associated with pelvic inflammatory disease and bacterial vaginosis [2]. *Ureaplasma *species can cause acute urethritis and have been associated with bacterial vaginosis, preterm birth, and neonatal respiratory disease [1, 3].

 Although *M. hominis* and *Ureaplasma *species can be cultured, this requires technical skill for interpretation of microscopic colonies and takes two to five days. *U. urealyticum *was the only *Ureaplasma* species until 2002, when *U. parvum* was described [4]. The two are not distinguished based on culture characteristics alone. Real-time PCR detection of these microorganisms from clinical samples circumvents technical issues related to culture and shortens turn-around time for detection and identification.

Few real-time PCR assays and associated studies have been described for *M. hominis. *A real-time PCR assay targeting *M. hominis gap* identified two positive cervical swabs from women being evaluated for infertility [5]. 153 urogenital specimens were tested with a real-time PCR assay targeting *M. hominis yid*C, of which 45 were PCR- and culture positive and 10 PCR positive only [6]. Finally, extragenital *M. hominis *infection was diagnosed in three patients using a real-time PCR assay targeting the *M. hominis *16S ribosomal RNA gene [7].

There has been more work on real-time PCR assays for *Ureaplasma *species, although some have described assays but have not evaluated clinical specimens or clinical isolates [8]. A real-time PCR assay that detects and distinguishes *U. urealyticum *from *parvum *was described but used to assess 87 vaginal swabs [9]. Tang et al. used a real-time PCR assay that detects and distinguishes *U. parvum *and *urealyticum* to test 346 genitourinary swabs; 120 were positive for the former and 21 for the latter, including 5 positive for both [10]. Finally, Vancutsem et al. used a real-time PCR assay for detection and differentiation of *U. urealyticum *and *parvum *to evaluate 300 lower genital tract specimens; 132 were culture positive, of which all plus an additional 19 were PCR-positive (19, *U. urealyticum; *120, *U. parvum;* 12, *Ureaplasma *species) [11]. 

Herein, we present one real-time PCR assay for the detection of *M. hominis* and another for the detection and differentiation of *Ureaplasma *species and report results of these assays on 283 genitourinary specimens in comparison to culture.

## 2. Materials and Methods

### 2.1. Clinical Specimens

283 genitourinary specimens (swabs, urine) submitted to the Mayo Clinic Clinical Microbiology Laboratory in transport medium (e.g., UTM, M5) for *M. hominis* and *Ureaplasma *culture were evaluated. No clinical data associated with these specimens was available. This study was approved by the Mayo Clinic Institutional Review Board.

### 2.2. *Mycoplasma hominis* Culture

Samples were placed into arginine broth, incubated at 35°C, and monitored four times daily for up to five days. Color change (indicating an alkaline pH shift) in the arginine broth prompted subculture of 50 *μ*L to an A7 agar plate. Plates were incubated anaerobically at 35°C for up to five days and examined daily with an inverted light microscope for “fried egg” morphology colonies.

### 2.3. *Ureaplasma* Culture

Samples were placed into U9 broth, incubated at 35°C, and monitored four times daily for up to five days. A color change (indicating an alkaline pH shift) in the U9 broth prompted subculture of 100 *μ*L to an A7 agar plate. Plates were incubated anaerobically at 35°C for up to 48 hours and examined with an inverted light microscope for small, circular to irregular colonies growing into the surface of the agar, with a surrounding red zone. Confirmation of *Ureaplasma *species was indicated by golden-brown stained colonies with the addition of 0.167 M CO(NH_2_)_2_ and 0.04 M MnCl_2_ in water.

### 2.4. Sample Processing for PCR

Samples were vortexed and 200 *μ*L transferred to a MagNA Pure sample cartridge (Roche Applied Science, Indianapolis, IN). DNA extraction was performed on the MagNA Pure LC 2.0 using the MagNA Pure LC Total Nucleic Acid Isolation Kit (Roche Applied Science) with a final elution volume of 100 *μ*L.

### 2.5. Polymerase Chain Reaction Assay

Primers and probes (Table 1) were designed using the LightCycler Probe Design Software, version 2.0 (Roche Diagnostics, Indianapolis, IN, USA) and DNA Workbench, version 5.7.1 (CLC Bio, Cambridge, MA, USA). Positive control plasmids were constructed for the three target-specific genes (Table 1) using the pCR 2.1 TOPO TA Cloning Kit (Invitrogen Corporation, Carlsbad, CA, USA). Sources for the inserted target sequences were *M. hominis *ATCC 23114, *U. urealyticum *ATCC 27618, and *U. parvum *ATCC 27815D. Plasmids were purified using the High Pure Plasmid Isolation Kit (Roche Applied Science). Sizes of the cloned inserts were confirmed by *Eco*R1 digestion. Plasmid inserts were sequenced using M13 forward and reverse primers included in the cloning kit, to confirm proper insert orientation. Plasmids were diluted in Tris-EDTA buffer (pH 8.0) and stored at 4°C.

The two assays were independently optimized on the LightCycler 480 II platform employing LightCycler 480 Software version 1.5 (Roche Applied Science). 15 *μ*L of PCR master mix, containing final concentrations of 1X Roche Genotyping Master (*Taq* DNA polymerase, PCR reaction buffer, deoxyribonucleoside triphosphate with dUTP substituted for dTTP and 1 mM MgCl_2_), 1 mM (additional) MgCl_2_, and 1X of each of the LightCycler primer-probe sets (Table 1) were added to a 96-well LightCycler 480 plate. Extracted nucleic acid (5 *μ*L) was then added to each well. The cycling program was as follows: denaturation at 95°C for 10 min; amplification for 45 cycles of 10 s at 95°C, 15 s at 55°C (single acquisition), and 15 s at 72°C; melting curve analysis for 30 s at 95°C, 10 s at 59°C, 15 s at 45°C (ramp rate of 0.1°C/s), and 0 s at 80°C (ramp rate of 0.14°C/s and continuous acquisition); and cooling for 30 s at 40°C. Positive and negative controls were included in each run. The positive control consisted of the abovementioned plasmids in S.T.A.R. buffer : sterile water (1 : 1) at a concentration of 1,000 targets/*μ*L. The negative control consisted of 1,000 colony forming units of *Escherichia coli* ATCC 25922 S.T.A.R. buffer : sterile water (1 : 1) at a concentration of 1,000 targets/*μ*L.

### 2.6. Polymerase Chain Reaction Sensitivity and Specificity

Predicted amplified product, primer, and probe sequences were subjected to BLAST searches using the National Center for Biotechnology Information (NCBI) genomic database (http://www.ncbi.nlm.nih.gov/). Analytical sensitivity was assessed by spiking a series of six tenfold dilutions of quantified genomic DNA from *M. hominis* ATCC 23114, *U. urealyticum* ATCC 27816, and *U. parvum* ATCC 27815D into genitourinary samples. Each dilution was extracted in triplicate and each extract was assayed in duplicate. The limit of detection was the lowest dilution where all six replicates were detected. Inclusivity and cross-reactivity were assessed using a panel organisms (Table 2), including 16 members of the Mollicutes class.

Clinical sensitivity and specificity were assessed by assaying the aforementioned clinical specimens and comparing results to those of culture. Discordant samples were tested courtesy of Dr. Stellrecht, at an independent clinical laboratory (Albany Medical Center) with a previously described assay [12]. 

**Figure 1:**
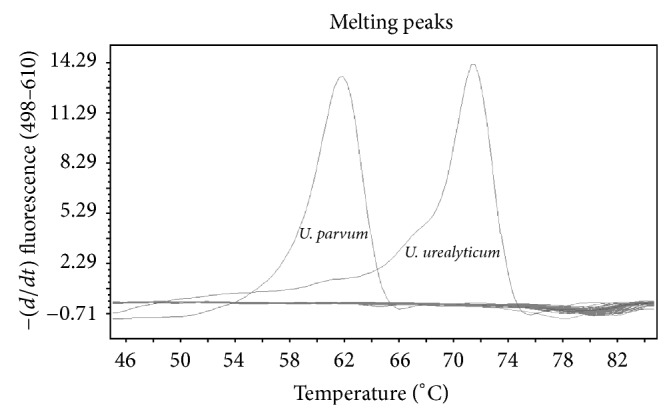
Melting curves of *Ureaplasma urealyticum* and *Ureaplasma parvum*.

**Figure 2:**
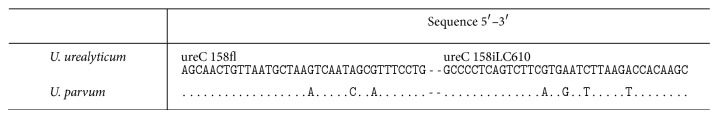
*Ureaplasma *probe design.

The ability of the *Ureaplasma *assay to differentiate *urealyticum *from *parvum *was assessed as follows. Cultured isolates from clinical samples were directly subjected to PCR with species differentiation based on melting curve analysis; sequence variations underlying the probed regions of *U. urealyticum* and* parvum* result in separation of the melting temperature of the two species (Figures 1 and 2). Results were compared to those of a previously described conventional PCR speciation method targeting the multiple-banded antigen using primers UMS-57 and UMA222 for *U. parvum* and UMS-170 and UMA263 for *U. urealyticum* [13].

### 2.7. Statistical Analysis

Assessment of the assays' sensitivity and specificity, with associated 95% confidence intervals (CI), compared to that of culture for *M. hominis *and *Ureaplasma *species was made using SAS software version 9.1 (SAS, INC, Cary, NC, USA). 

## 3. Results

### 3.1. Polymerase Chain Reaction Sensitivity and Specificity

The analytical sensitivity of both assays was 100 genome copies/*μ*L genitourinary specimen. Amplified product, primer, and probe sequences were subjected to NCBI database searches using BLAST software; no significant homology was noted outside of the genera targeted by these assays. Nucleic acid material from members of the Mollicutes class, excluding *M. hominis* and the *Ureaplasma *species, was not detected (Table 2).

### 3.2. Clinical Sensitivity and Specificity

The *M. hominis *PCR assay had a clinical sensitivity and specificity of 90.7% (95% CI: 77.4%, 97.3%) and 99.2% (95% CI: 97.0%, 99.9%), respectively (Table 3). The 6 discordant results were tested at the Albany Medical Center using an assay targeting the 16S ribosomal RNA gene; [12] both PCR positive/culture-negative specimens were PCR positive, and three of four PCR negative/culture-positive specimens were PCR negative.

The *Ureaplasma *PCR assay had a clinical sensitivity and specificity of 96.5% (95% CI: 92.1%, 98.9%) and 93.8% (95% CI: 88.1%, 97.0%), respectively (Table 3). The 14 discordant results were tested at Albany Medical Center; [12] five of nine specimens that were PCR positive/culture negative were PCR positive, and all five specimens that were PCR negative/culture positive were PCR negative. Of the specimens that tested positive for *Ureaplasma *species by PCR and were culture positive, *U. urealyticum *alone was detected in 28, *U. parvum* alone in 109, and both in 2. Among the PCR positive/culture-negative specimens, *U. urealyticum* was detected in 3 and *U. parvum* in 6.

Thirty-one culture isolates of *Ureaplasma *species were tested with the *Ureaplasma* assay and a previously reported PCR method that differentiates between the two species [13]. The reference method yielded species-level identification for 20 isolates, including 4 *U. urealyticum *and 16 *U. parvum*, with identical results to the assay described herein. The remaining 11 isolates were speciated by the assay described herein but not by the reference method; they were confirmed to be *Ureaplasma *species by partial 16S ribosomal RNA gene sequencing [14]. All partial 16S ribosomal RNA gene sequences were identical to one another and were perfect matches to bases 145,365 through 145,845 of GenBank AF222894.1 (*U. parvum*) and bases 40 through 520 of GenBank L08642.1 (*U. urealyticum*).

## 4. Discussion

We describe two rapid real-time PCR assays, one for detection of *M. hominis *and the other for detection of *Ureaplasma *species; they have comparable performance to culture but yield results in three hours, instead of two to five days for culture. These assays are performed on a standard platform and are adaptable to automation, a potential advantage over other described methods, especially for large reference laboratories that process large numbers of specimens. 

We are not aware of other real-time PCR studies that have assessed *M. hominis *and *Ureaplasma *species using the same set of clinical samples. Overall, 14% of tested specimens were PCR positive for *M. hominis *and 52% for *Ureaplasma *species. A multiplex PCR enzyme-linked immunosorbent assay was used to detect *M. hominis* and *U. parvum* and *urealyticum* in cervical swabs from 175 Australian women with and without cervicitis; 16% tested positive for *M. hominis* and 68% for *Ureaplasma *species [15]. Multiplex PCR and autocapillary electrophoresis were used to detect *M. hominis *and *Ureaplasma *species (without differentiating *U. parvum *from *urealyticum*) in genitourinary specimens from 113 South Koreans with sexually transmitted infections; 12% were positive for *M. hominis* and 43% for *Ureaplasma *species [16]. These findings are similar to ours [15, 16].

Our PCR assay not only detects *Ureaplasma *species but also differentiates *U. parvum *from *urealyticum. *As in prior studies, *U. parvum *was more common than *U. urealyticum*, [10, 11, 15, 17] with 41% of the genitourinary specimens testing positive for the former and 12% for the latter. In one prior study, 63% of specimens were positive for *U. parvum *and 7% for *U. urealyticum* [15]. Another study showed, using a multiplex PCR-reverse line blot assay, that 48% of first voided urine specimens from women attending sexual health clinics in Australia were positive for *U. parvum *and 25% for *U. urealyticum* [17]. In the study by Tang et al., 36% of genitourinary swabs collected from hospitalized males and females in China were positive for *U. parvum *and 8% for *U. urealyticum* [10]. Finally, in study by Vancutsem et al., 44% of lower genital tract specimens obtained from healthy women at their first prenatal visit in Belgium were positive for *U. parvum *and 10% for *U. urealyticum* [11]. Despite different geographic locales and clinical status, these numbers are strikingly similar.

In addition to the advantage of speed, the described assays overcome the challenges of detection of these organisms by culture. Although culture is considered a gold standard method (and was so considered in this study), colonial identification is challenging and subjective because it is done using the human eye and a dissecting microscope. Artifacts may be misidentified as colonies, yielding false-positive results, or colonies may be overlooked, yielding false-negative results. Although PCR may be considered more technically complex, in a laboratory where technologists are familiar with PCR, this approach is more user-friendly (and generalizable among assays for various microorganisms) than culture.

The described assays may be useful for investigating epidemiology and pathogenesis of infections with *U. parvum *and *urealyticum* [2, 18]. Although extra-genital specimens were not tested, the described *M. hominis *assay may be useful to detect extra-genital *M. hominis *infections [7].

## Figures and Tables

**Table 1:** Primers and probes.

*Mycoplasma hominis * *tuf* (set number 793, TIB MolBio, Aldelphia, NJ; 10X concentration)^a^		
Primers	*tuf* 193F	5′ AATTGATATGTTTAAAGATGATGAAAGAGA 3′
	*tuf* 193R	5′ TGTATCAACAGCATCCATTAATTCC 3′
Probes	*tuf* 193fl^b^	5′ GACGTAAGAAGCCTTCTATCAGAATATGGT FITC 3′
	*tuf* 193iLC610^c^	5′ Red610 TGATGGTGACAATGCTCCTATTATTGCTGGTTC PO4 3′

*Ureaplasma *species *ure*C (set number 684, TIB MolBio; 10X concentration)^d^		

	*ure*C 158F	5′ CCTGCTTCGTTTAATGTATCTG 3′
Primers	*ure*C 158R	5′ GAAGATCCAATCTTTGAACAAATCGTA 3′
	*ure*C 158R5	5′ GAAGATCCAATCTTTGAACAAATTGCT 3′
Probes	*ure*C 158fl^b^	5′ AGCAACTGTTAATGCTAAGTCAATAGCGTTTCCTG FITC 3′
	*ure*C 158iLC610^c^	5′ Red610 GCCCCTCAGTCTTCGTGAATCTTAAGACCACAAGC PO4 3′

^a^ *tuf* target corresponds to 66720–66912 of GenBank accession number FP236530.

^b^Labeled with fluorescein on 3′ end.

^c^Labeled with LC610 on 5′ end and a phosphate on 3′ end.

^d^ *ure*C target corresponds to 527786–527943 of GenBank accession number CP001184.

**Table 2:** Cross-reactivity and inclusivity panel. Only *Mycoplasma hominis, Ureaplasma parvum*,  and *Ureaplasma urealyticum *were detected (by the appropriate assays).

Organism	Accession no. or source	Organism	Accession no. or source
*Acholeplasma laidlawii *	ATCC 23206	*Entamoeba histolytica *	ATCC 30459
*Acinetobacter baumannii *	ATCC 19606	*Entamoeba moshkovskii *	ATCC 30042
*Acinetobacter lwoffii/haemolyticus *	QC Strain	*Enterobacter cloacae *	ATCC 13047
*Actinomyces odontolyticus *	ATCC 17929	*Enterococcus faecalis *	ATCC19433U
*Aeromonas hydrophila *	CAP-D-1-82	*Enterococcus faecium *	ATCC 19434
*Arcanobacterium haemolyticum *	ATCC 9345	*Escherichia coli *	ATCC 25922
*Arcanobacterium pyogenes *	ATCC 19411	*Escherichia coli* O142:K86(B):H6	ATCC 23985
*Parabacteroides distasonis *	ATCC 8503	*Escherichia coli *O157:H7	ATCC 35150
*Bacteroides fragilis *	ATCC 25285	*Escherichia coli *O70:K:H42	ATCC 23533
*Bacteroides thetaiotaomicron *	ATCC 29741	*Escherichia fergusonii *	ATCC 35469
*Bacteroides vulgatus *	ATCC 29327	*Escherichia hermannii *	ATCC 33650
*Bifidobacterium adolescentis *	ATCC 15703	*Escherichia vulneris *	ATCC 33821
*Bifidobacterium bifidum *	ATCC 29521	*Eubacterium rectale *	ATCC 33656
*Bordetella bronchiseptica *	ATCC 19395	*Finegoldia magna *	ATCC 29328
*Bordetella holmesii *	ATCC 51541	*Fluoribacter bozemanae *	ATCC 33217
*Bordetella parapertussis *	ATCC 15311	*Fluoribacter gormanii *	ATCC 33297
*Bordetella pertussis *	ATCC 9797	*Fusobacterium nucleatum *	ATCC 25559
*Burkholderia cepacia *	SCB1277	*Gardnerella vaginalis *	NYS 4-87
*Campylobacter coli *	ATCC 33559	*Giardia lamblia *	ATCC 30957
*Campylobacter jejuni *	ATCC 33560	*Haemophilus influenzae *	ATCC 10211
*Chlamydia trachomatis *	ATCC VR-348B	Human DNA	MRC-5 cells
*Chlamydophila pneumoniae *	ATCC 53592	*Klebsiella oxytoca *	ATCC 700324
*Chlamydophila pneumoniae *	ATCC VR-1310	*Klebsiella pneumoniae *	ATCC 700603
*Citrobacter freundii *	ATCC 8090	*Lactobacillus delbrueckii *ssp. *lactis *	ATCC 12315
*Clostridium difficile *	ATCC 9689	*Lactobacillus rhamnosus *	ATCC 7469
*Clostridium perfringens *	ATCC 13124	*Fluoribacter dumoffii *	ATCC 33279
*Clostridium ramosum *	ATCC 25582	*Legionella jordanis *	ATCC 33623
*Collinsella aerofaciens *	ATCC 25986	*Legionella longbeachae *	ATCC 33462
*Corynebacterium diphtheriae *	SCB-25-86	*Tatlockia micdadei *	ATCC 33204
*Corynebacterium pseudodiphtheria *	NY-4-88	*Legionella pneumophila *	ATCC 33152
*Cryptosporidium *species	feline isolate	*Legionella wadsworthii *	ATCC 33877
*Dientamoeba fragilis *	ATCC 30948	*Listeria monocytogenes *	ATCC 15313
*Eggerthella lenta *	ATCC 25559	*Moraxella catarrhalis *	ATCC 8176
*Encephalitozoon cuniculi *	JS strain	*Morganella morganii *	CAP-D-5-79
*Encephalitozoon hellem *	ATCC 50451	*Mycobacterium africanum *	ATCC 25420
*Encephalitozoon intestinalis *	ATCC 50651	*Mycobacterium avium *	ATCC 700398
*Mycobacterium avium *	ATCC 700897	*Proteus mirabilis *	ATCC 35659
*Mycobacterium bovis *	ATCC 19210	*Proteus vulgaris *	QC strain
*Mycobacterium bovis *(BCG)	ATCC 35735	*Pseudomonas aeruginosa *	ATCC 27853
*Mycobacterium gordonae *	ATCC 14470	*Pseudomonas fluorescens/putida *	CDC-AB4-B10-84
*Mycobacterium intracellulare *	ATCC 35761	*Rhodococcus equi *	ATCC 6939
*Mycobacterium kansasii *	ATCC 12478	*Salmonella enterica *	ATCC 35987
*Mycobacterium microti *	ATCC 19422	*Salmonella* serogroup B	CAP-D-1-69
*Mycobacterium smegmatis *	ATCC 19980	*Shigella dysenteriae *	CDC 82-002-72
*Mycobacterium tuberculosis *	ATCC 25177	*Shigella flexneri *serotype 2a	ATCC29903
*Mycobacterium tuberculosis *	ATCC 27294	*Shigella sonnei *	ATCC 25931
*Mycobacterium tuberculosis *	ATCC 35825	*Staphylococcus aureus *	ATCC 25923
*Mycobacterium tuberculosis *	ATCC 35837	*Staphylococcus epidermidis *	ATCC 14990
*Mycoplasma arginini *	ATCC 23838D	*Stenotrophomonas maltophilia *	SCB-33-77
*Mycoplasma arthritidis *	ATCC 19611D	*Streptococcus bovis *	CAP-D-16-83
*Mycoplasma bovis *	ATCC 25523D	*Streptococcus pneumoniae *	ATCC 49619
*Mycoplasma buccale *	ATCC 23636	*Streptococcus pyogenes *	ATCC 19615
*Mycoplasma faucium *	ATCC 25293	*Streptococcus sanguinis *	ATCC 10556
*Mycoplasma fermentans *	ATCC 19989	∗ *Ureaplasma parvum *	ATCC 28715
*Mycoplasma genitalium *	ATTC 33530	∗ *Ureaplasma urealyticum *	ATCC 27618
∗ *Mycoplasma hominis *	ATCC 23114	*Yersinia enterocolitica *	ATCC 9610
*Mycoplasma hyorhinis *	ATCC 17981D	BK polyomavirus	ATCC VR-837
*Mycoplasma lipophilum *	ATCC 27104	Cytomegalovirus	ATCC VR-538
*Mycoplasma orale *	ATCC 23714		
*Mycoplasma phocidae *	ATCC 33657	Herpes simplex virus 1	Lab Control
*Mycoplasma pirum *	ATCC 25960D	Herpes simplex virus 2	Lab Control
*Mycoplasma pneumoniae *	ATCC 15531D	Human adenovirus 9	ATCC VR-1086
*Mycoplasma salivarium *	ATCC 23064	Human coronavirus 229E	ATCC VR-740
*Neisseria gonorrhoeae *	ATCC 43069	Human coxsackievirus B 1 (*Enterovirus*)	ATCC VR-28
*Neisseria lactamica *	ATCC 23970	Human herpesvirus 6B	ATCC VR-1467
*Neisseria meningitidis *	ATCC 13077	Human herpesvirus 7	ABI 08765000
*Nocardia brasiliensis *	ATCC 51512	Human herpesvirus 8	ABI 08735000
*Nocardia brevicatena *	ATCC 15333	Human parainfluenza virus 1	ATCC VR-94
*Nocardia carnea *	ATCC 6847	Human parainfluenza virus 3	ATCC VR-93
*Nocardiopsis dassonvillei *	ATCC 23218	Respiratory syncytial virus A2	ATCC VR-1540
*Nocardia farcinica *	ATCC 3318	Respiratory syncytial virus B	ATCC VR-1401
*Nocardia otitidiscaviarum *	ATCC 14629	Influenza A virus (H3N2)	ATCC VR-810
*Nocardia transvalensis *	ATCC 6865	Influenza B virus	ATCC VR-791
*Plesiomonas shigelloides *	ATCC 14029	Measles virus	ATCC VR-24
*Porphyromonas gingivalis *	ATCC 33277	Mumps virus	ATCC VR-365
*Prevotella melaninogenica *	ATCC 25845	Varicella-zoster virus	ATCC VR-1367
*Prevotella oralis *	ATCC 33269		

**Table 3:** Comparison of PCR with culture for *Mycoplasma hominis *and *Ureaplasma *species detection.

		*M. hominis* culture		
		Positive	Negative	
*M. hominis *PCR	Positive	38	2	40
	Negative	4	239	243
		42	241	283

Sensitivity = 90.5% (95% CI: 77.4%, 97.3%), specificity = 99.2% (95% CI: 97.0%, 99.9%)				

		*Ureaplasma* species culture		
		Positive	Negative	

*Ureaplasma *PCR	Positive	139^1^	9^2^	148
	Negative	5	130	135
		144	139	283

Sensitivity = 96.5% (95% CI: 92.1%, 98.9%), specificity = 93.5% (95% CI: 88.1%, 97.0%)				

^1^ *U. urealyticum* (*n* = 28), *U. parvum* (*n* = 109), *U. urealyticum* and *U. parvum* (2).

^2^ *U. urealyticum* (*n* = 3), *U. parvum* (*n* = 6).

## References

[1] Taylor-Robinson D., Jensen J. S. (2011). *Mycoplasma genitalium*: from chrysalis to multicolored butterfly. *Clinical Microbiology Reviews*.

[2] Patel M. A., Nyirjesy P. (2010). Role of *Mycoplasma* and *Ureaplasma* species in female lower genital tract infections. *Current Infectious Disease Reports*.

[3] Aaltonen R., Jalava J., Laurikainen E., Kärkkäinen U., Alanen A. (2002). Cervical *Ureaplasma urealyticum* colonization: comparison of PCR and culture for its detection and association with preterm birth. *Scandinavian Journal of Infectious Diseases*.

[4] Robertson J. A., Stemke G. W., Davis J. W. (2002). Proposal of *Ureaplas maparvum* sp. nov. and emended description of *Ureaplasma urealyticum* (Shepard et al. 1974) Robertson et al. 2001. *International Journal of Systematic and Evolutionary Microbiology*.

[5] Baczynska A., Svenstrup H. F., Fedder J., Birkelund S., Christiansen G. (2004). Development of real-time PCR for detection of *Mycoplasma hominis*. *BMC Microbiology*.

[6] Férandon C., Peuchant O., Janis C. (2011). Development of a real-time PCR targeting the *yidC* gene for the detection of *Mycoplasma hominis* and comparison with quantitative culture. *Clinical Microbiology and Infection*.

[7] Pascual A., Jaton K., Ninet B., Bille J., Greub G. (2010). New diagnostic real-time PCR for specific detection of *Mycoplasma hominis* DNA. *International Journal of Microbiology*.

[8] Xiao L., Glass J. I., Paralanov V. (2010). Detection and characterization of human *Ureaplasma* species and serovars by real-time PCR. *Journal of Clinical Microbiology*.

[9] Yi J., Bo H. Y., Kim E. C. (2005). Detection and biovar discrimination of *Ureaplasma urealyticum* by real-time PCR. *Molecular and Cellular Probes*.

[10] Tang J., Zhou L., Liu X., Zhang C., Zhao Y., Wang Y. (2011). Novel multiplex real-time PCR system using the SNP technology for the simultaneous diagnosis of *Chlamydia trachomatis*, *Ureaplasma parvum* and *Ureaplasma urealyticum * and genetic typing of serovars of *C. trachomatis* and *U. parvum* in NGU. *Molecular and Cellular Probes*.

[11] Vancutsem E., Soetens O., Breugelmans M., Foulon W., Naessens A. (2011). Modified real-time PCR for detecting, differentiating, and quantifying *Ureaplasma urealyticum* and *Ureaplasma parvum*. *Journal of Molecular Diagnostics*.

[12] Stellrecht K. A., Woron A. M., Mishrik N. G., Venezia R. A. (2004). Comparison of multiplex PCR assay with culture for detection of genital mycoplasmas. *Journal of Clinical Microbiology*.

[13] Kong F., Ma Z., James G., Gordon S., Gilbert G. L. (2000). Species identification and subtyping of *Ureaplasma parvum* and *Ureaplasma urealyticum* using PCR-based assays. *Journal of Clinical Microbiology*.

[14] Baracaldo T., Foltzer M., Patel R., Bourbeau P. (2012). Empyema caused by *Mycoplasma salivarium*. *Journal of Clinical Microbiology*.

[15] McIver C. J., Rismanto N., Smith C. (2009). Multiplex PCR testing detection of higher-than-expected rates of cervical *Mycoplasma*, *Ureaplasma*, and *Trichomonas* and viral agent infections in sexually active australian women. *Journal of Clinical Microbiology*.

[16] Samra Z., Rosenberg S., Madar-Shapiro L. (2011). Direct simultaneous detection of 6 sexually transmitted pathogens from clinical specimens by multiplex polymerase chain reaction and auto-capillary electrophoresis. *Diagnostic Microbiology and Infectious Disease*.

[17] McKechnie M. L., Hillman R. J., Jones R. (2011). The prevalence of urogenital micro-organisms detected by a multiplex PCR-reverse line blot assay in women attending three sexual health clinics in Sydney, Australia. *Journal of Medical Microbiology*.

[18] Barykova LD Y. A., Shmarov M. M., Vinarov A. Z. (2011). Association of *Mycoplasma hominis* infection with prostate cancer. *Oncotarget*.

